# Fine-scale population structure and ecotypes of anadromous Hilsa shad (*Tenualosa ilisha*) across complex aquatic ecosystems revealed by NextRAD genotyping

**DOI:** 10.1038/s41598-019-52465-2

**Published:** 2019-11-05

**Authors:** Md Asaduzzaman, Md A. Wahab, Md J. Rahman, Md Nahiduzzzaman, Malcom W. Dickson, Yoji Igarashi, Shuichi Asakawa, Li Lian Wong

**Affiliations:** 1 Department of Marine Bioresource Science, Faculty of Fisheries, Chattogram Veterinary and Animal Sciences University, Khulshi 4225, Chattogram, Bangladesh; 2 WorldFish, Bangladesh and South Asia Office, Banani, Dhaka, 1213 Bangladesh; 3 Laboratory of Aquatic Molecular Biology and Biotechnology, Department of Aquatic Bioscience, The University of Tokyo, 1-1-1 Yayoi, Bukkyo-ku, Tokyo 113-8657 Japan; 4 Institute of Tropical Aquaculture and Fisheries Research, Universiti Malaysia Terengganu, 21030 Kuala, Terengganu Malaysia; 5 Institute of Marine Biotechnology, Universiti Malaysia Terengganu, 21030 Kuala, Terengganu Malaysia

**Keywords:** Molecular ecology, Population genetics

## Abstract

The anadromous Hilsa shad (*Tenualosa ilisha*) live in the Bay of Bengal and migrate to the estuaries and freshwater rivers for spawning and nursing of the juveniles. This has led to two pertinent questions: (i) do all Hilsa shad that migrate from marine to freshwater rivers come from the same population? and (ii) is there any relationship between adults and juveniles of a particular habitat? To address these questions, NextRAD sequencing was applied to genotype 31,276 single nucleotide polymorphism (SNP) loci for 180 individuals collected from six strategic locations of riverine, estuarine and marine habitats. F_ST_ OutFLANK approach identified 14,815 SNP loci as putatively neutral and 79 SNP loci as putatively adaptive. We observed that divergent local adaptations in differing environmental habitats have divided Hilsa shad into three genetically structured ecotypes: turbid freshwater (Western Riverine), clear freshwater (Eastern Riverine) and brackish-saline (Southern Estuarine-Marine). Our results also revealed that genes involved in neuronal activity may have facilitated the juveniles’ Hilsa shad in returning to their respective natal rivers for spawning. This study emphasized the application of fundamental population genomics information in strategizing conservation and management of anadromous fish such as Hilsa shad that intersect diverse ecotypes during their life-history stages.

## Introduction

Anadromous fish migrate from a freshwater breeding habitat to a marine feeding habitat and back to freshwater for spawning and their level of genetic diversity and population divergence is intermediate to that of marine and freshwater species^1–3^. The Hilsa shad (*Tenualosa ilisha*, Hamilton, 1822) is an anadromous clupeid species with a diverse range of distribution extending from freshwater rivers, estuaries, foreshore areas and seas of the Indo-Pacific region. It is the most important commercial trans-boundary species of which Bangladesh has the major share (86%), followed by India (8%), Myanmar (4%) and other countries within the Bay of Bengal region^4^. Hilsa provides the nutrition of 260 million Bengali people living in Bangladesh, some states of India and others living around the world, and it also support the livelihood of 2.5 million fishers and related people in the value chain alone in Bangladesh^5^. It is the single-most dominating food fish in the Bay of Bengal region with an average of 44% contribution to capture fisheries in Bangladesh^6^.

The Hilsa shad mainly inhabits in the Bay of Bengal including lower regions of the estuaries and foreshore areas, but migrates to the upstream rivers, mainly the Ganges-Brahmaputra-Meghna river systems during the spawning season and returns to the original habitat after spawning^7^. There are some exceptions to this behavior as two other subtypes of the species -a marine type and a fluvial potamodromous type have also been reported^8–10^. The marine subtype inhabited nearshore coastal and/or sea habitats and relies on downstream estuarine waters for spawning without migrating to freshwater^9–11^. The potamodromous types appear to stay in the middle reaches of the major river system and complete its life cycle within freshwater without migrating to the sea^8,10^. However, it is not truly known whether the anadromous *T. ilisha* mix and breed with two others minor subtypes during migration or whether they pass each other spatially and temporally. Therefore, understanding of stock structure and divergence in Hilsa shad population across its range of different habitats (sea, estuary and rivers) is still in dispute.

Understanding the genetic background and population structure of a species is crucial for planning and implementing conservation and management schemes, and needs to be explicitly considered for long-term sustainability. It is highly essential for fishery managers to know whether they are dealing with single or multiple spawning populations to design a sustainable management plan. However, revealing the genetic structure of an anadromous fish is challenging for conservation and management purposes^12^. This is mainly because of the highly connected diverged population with large effective population size of anadromous fish often show very weak genetic differentiation, thus decreasing the power of genetic tools in assigning individuals to their origins and defining the management units. However, the recent advances in next-generation sequencing (NGS) genotyping methods have expanded the prospects for exploring adaptive genetic markers to finely define weakly structured population, which ultimately improve our understanding of the genetic basis of fitness traits^13^.

Past research on the population genetic structure of this Hilsa shad mostly focused to investigate whether or not the species belongs to a single stock that uses rivers, brackish water estuaries and marine waters. The previous studies were mostly conducted through the use of allozyme markers^14–16^, Restriction Fragment Length Polymorphism (RFLP)^17,18^, Random Amplification of Polymorphic DNA (RAPD) markers^19–22^, and mitochondrial DNA cytochrome *b* gene nucleotide sequencing^23,24^. However, results from all of these studies are contradictory and inconclusive. The allozyme marker based studies reported that there is only one overall panmictic population of Hilsa shad in the Gangetic river systems^15^ and the Bay of Bengal region including Bangladesh, India and Myanmar^16^, but the Hilsa shad of the Bay of Bengal was genetically distinct from Kuwait and Indonesian Hilsa shad population^16^. Similarly, study-using mitochondrial DNA cytochrome *b* region also reported that the Hilsa shad population of the Ganga and the Hoogly rivers are the same population^23^. In contrast, studies using RAPD markers indicated that there is more than one gene pool of Hilsa shad in Bangladesh waters^19,20^. Similarly, it was reported that Hilsa shad has genetically two different populations from two major inland rivers of Bangladesh, the Padma and the Meghna^20^. PCR-RFLP analysis of the mitochondrial DNA D-loop region provided the same conclusion, suggesting that the Hilsa shad population has at least two differentiated populations in Bangladesh waters, which were subsequently divided into three, corresponding to the riverine, estuarine and marine populations^18^.

The inconclusive results of the past research about the population structure of the Hilsa shad can be addressed by using the genome-wide approach, which allows the genotyping of thousands of markers to detect genetic structure of this species at a finer spatial scale^25^. NGS-based restriction-site-associated DNA (NextRAD) techniques is a modern approach of high-throughput sequencing techniques to study population genetic structures that simultaneously facilitate both genotyping-by sequencing and discovering a large number of single-nucleotide polymorphisms (SNPs) across the genome^26^. The NextRAD approach includes surveys of both neutral and adaptive panels of SNP loci that can be used to determine the levels of genetic differentiation and genetic diversity. This approach has already been successfully applied in several fish species and has proven to be effective at identifying the genomic basis of fitness traits^27–29^. Our application of nextRAD sequencing alleviates the problem of absent reference genome sequence of this non-model species and facilitates the development of SNP markers, with the aim of identification of candidate adaptive loci. The *F*_*ST*_ OutFLANK approach has been proven as a popular means for identifying the adaptive markers of a fish species in which annotated reference genome is not readily available^30,31^. As Hilsa shad is a highly dispersive, anadromous fish, represents an ideal candidate for identifying putatively adaptive panel of SNP loci with genome scan and outlier test.

Until today, two important questions about the population genetic structure and parental assignment remained unclear: (1) What is the degree of genetic differences among the Hilsa shad populations available in different migratory routes of the species? (2) Are the juveniles of Hilsa shad (locally known as *Jatka*) of a particular habitat return to their respective natal rivers (rivers where they were born/nursed) for spawning as adults? To unravel the answers of the above two questions, the present study was conducted by identifying a set of neutral and adaptive genetic markers. The NextRAD sequencing technique was employed to genotype 31,276 SNP loci for 180 individuals of the species collected from six strategic aquatic habitats for revealing parental assignment and stock structure of the Hilsa shad population in Bangladesh waters. We applied the *F*_*ST*_ OutFLANK approach to determine adaptive vs. neutral SNP loci, and additional analysis were employed to verify the significance of these putatively adaptive loci under selection in the context with different migratory routes and habitats.

## Material and Methodology

### Sample collection

**Figure 1:**
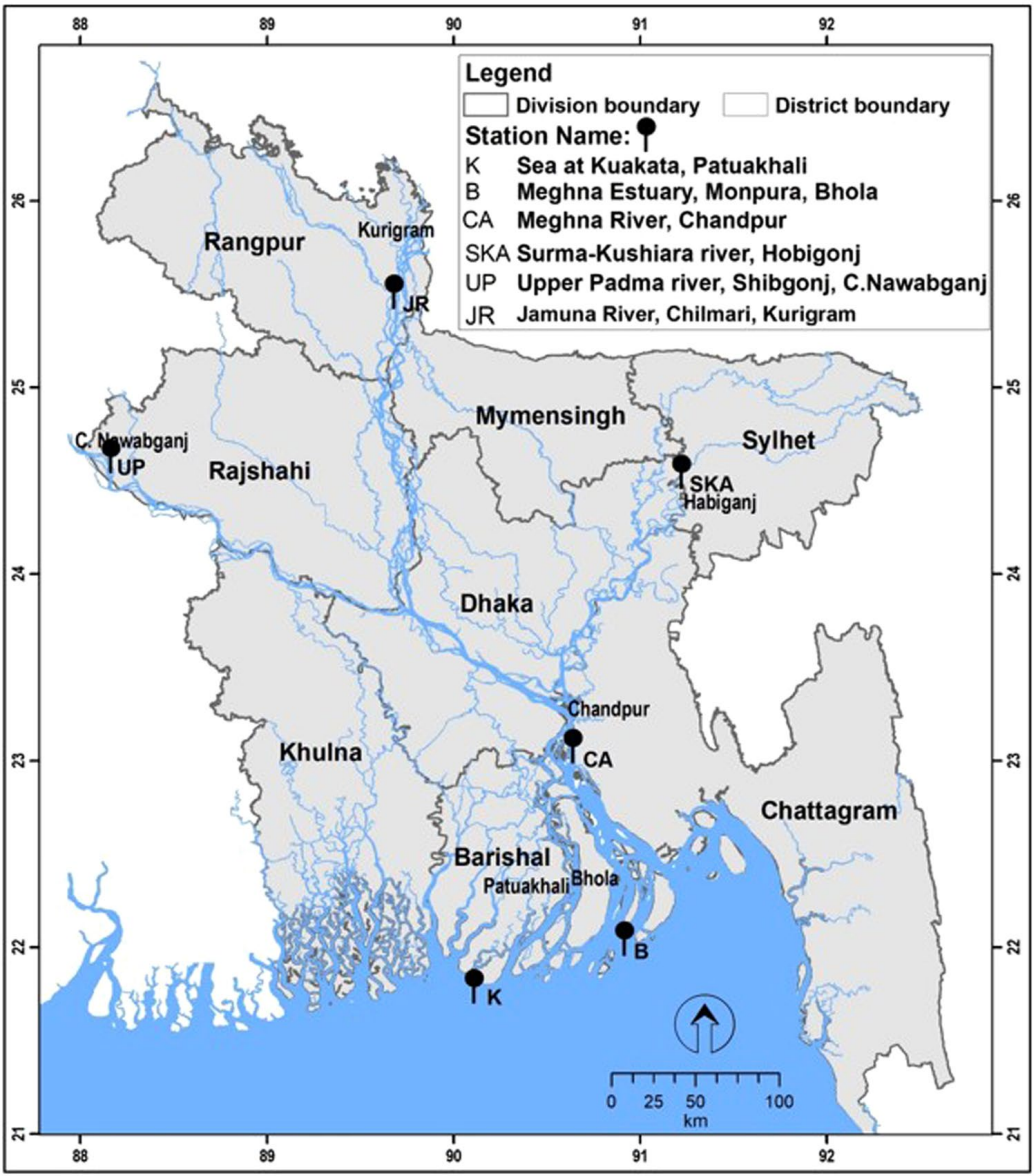
Info-map showing migration of three stocks of *Tenualosa ilisha* with their labels as in Table 1.

Juveniles and adults of *T. ilisha* were collected from the riverine, estuarine and coastal systems in Bangladesh, comprising six sampling sites (Fig. 1; Table 1). Two life stages were represented to address our research questions related to migration route and origins of these individuals with their respective populations. A total of 30 individuals were sampled from each population, with the life stages ratio dependent on their availability. Fin clips were preserved in absolute ethanol prior to DNA extraction. DNA isolations were performed using Promega DNA purification system (Promega, Madison, WI, USA) according to the manufacturer’s protocol. DNA quantifications were conducted using real-time PCR fluorescence measurements of double stranded DNA^32^ and the Quant-it kit (Life Technologies, Foster City, CA). All samples were collected under Bangladesh’s government permit and in accordance with animal care protocol (CVASU20160422) as approved by the Chattogram Veterinary and Animal Sciences University’s Animal Care and Biosafety Committee.

**Table 1:** Summary of sampling information for *Tenualosa ilisha* for nextRAD sequencing.

Site	Area	Year of collection	Latitude	Longitude	Sample sizes (N)	
					Juvenile	Adult
K	Sea at Kuakata Patuakhali	2017	21° 47′33.77″	90° 06′ 43.32″	15	15
B	Meghna Estuary Monpura, Bhola	2017	22° 02′ 53.41″	90° 55′ 07.65″	30	0
CA	Meghna River, Chandpur Sadar	2017	23° 04′ 49.91″	90° 38′ 28.53″	15	15
SKA	Surma-Kushiara River, Hobigonj	2016	24° 32′ 57.75″	91° 13′ 32.01″	26	4
UP	Upper Padma River, Shibgonj, Chapai Nawabganj	2016	24° 38′ 01.62″	88° 03′ 29.51″	21	9
JR	Jamuna River Chilmari, Kurigram	2017	25° 30′ 49.61″	89° 40′ 57.77″	18	12

### nextRAD genotyping

Genomic DNA was converted into nextRAD genotyping-by-sequencing libraries (SNPsaurus, LLC, USA). Genomic DNA was first fragmented with Nextera DNA Flex Library Prep Kit (Illumina, Inc, USA), which also ligates short adapter sequences to the ends of the fragments. The Nextera reaction was scaled for fragmenting 10 ng of genomic DNA, although 20 ng of genomic DNA was used for input to compensate for the amount of degraded DNA in the samples and to increase fragment sizes. Fragmented DNA was then amplified for 25 cycles at 72 °C, with one of the primers matching the adapter and extending 8 nucleotides into the genomic DNA with the selective sequence GTGTAGAG. Thus, only fragments starting with a sequence that can be hybridized by the selective sequence of the primer will be efficiently amplified. The nextRAD libraries were sequenced on a HiSeq 4000 with eight lanes of 150 bp reads (University of Oregon, USA). The resulting fragments are fixed at the selective end, and have random lengths depending on the initial Nextera fragmentation.

### Data quality control and filtering

Reads were trimmed using bbduk in custom scripts of SNPsaurus, LLC (BBMap tools, http://sourceforge.net/projects/bbmap/):bbmap/bbduk.shin = reads/run_1612/1612_GTAGAGGACTAAGCCT_S483_L006_R1_001_subset.fastq.gzout = reads/run_1612/1612_GTAGAGGACTAAGCCT_S483_L006_R1_001_t.fastq.gz ktrim = r k = 17 hdist = 1 mink = 8 ref = bbmap/resources/nextera.fa.gz minlen = 100 ow = t qtrim = r trimq = 10. The original shadstringent.vcf file contained data for 31, 276 SNP loci existing within a catalog of 92, 721 consensus NextRAD tagged sequences of 150 bases each. A column containing a unique ID for each SNP locus was added to the unfiltered vcf file using a custom perl script to remove redundancy among loci. Further filtering steps included the removal of complex SNPs with more than two alleles, less than 5% overall minor allele frequency, and less than 80% completeness of data among samples. Likewise, samples containing less than 80% completeness of data among the remaining loci were removed from the dataset. Additionally, where there were multiple SNPs located in a single NextRAD sequence tag only the first cataloged SNP was kept in the dataset so as to avoid the possibility of a single NextRAD locus having a disproportionate effect on the analyses. The unique IDs for SNP loci passing quality control standards were passed to a whitelist, which was used to filter the original vcf file using a custom perl script. After all filtering steps a total of 14, 894 individual SNP loci remained in the dataset. An additional custom perl script was used to convert the filtered vcf file to genepop format.

### SNP discovery and gene annotation

A de novo reference was created by collecting 10 million reads in total, evenly from the samples, and excluding reads that had counts of <7 or >1000. The remaining loci were then aligned to each other to identify allelic loci and collapse allelic haplotypes to a single representative. All reads were mapped to the reference with an alignment identity threshold of 0.95 using bbmap (BBMap tools). Genotype calling was done using Samtools and bcftools (samtools mpileup -gu -Q 15 -t DP, DPR -f ref.fasta -b samples.txt | bcftools call -cv - >genotypes.vcf). The vcf was filtered to remove alleles with a population frequency of <3%. Loci that were heterozygous in all samples or had more than 2 alleles in a sample (suggesting collapsed paralogs) were removed. The absence of artifacts was checked by counting SNPs at each read nucleotide position and determining that SNP number did not increase with reduced base quality at the end of the read. In addition, each haplotype from all nextRAD-tags that contained putatively adaptive loci were subject to a BLASTn^33^ search of all sequences in the NCBI non-redundant database (word size = 11; mismatch scores = 2, −3; maximum e-value = 15). To reduce annotations to repetitive sequences in the database, each locus in the vcf file had the associated sequence annotated by blast, using: blastn -db ncbi-blast-2.4.0+/db/nt and filtering for those blast hits with a significance of e-15 or better.

### Population genetic analysis

The filtered data was imported as a genind object into R and analyzed largely using the adegenet package^34^. After a first look revealed little variation among collections, an outlier locus approach was taken using the R package OUTFLANK^35^ which calculates a neutral distribution of Fst values and then uses this distribution to assign q-values to each locus to detect adaptive loci which are putatively influenced by selection. Default parameters were used for OUTFLANK analysis and the “number_of_samples” parameter was set to 6 (a number equal to the collections sampled). Output from this analysis was used to create a neutral loci dataset and an adaptive loci dataset for further analysis. The complete, outlier, and neutral datasets were further analyzed using the GenePop R package^36^. Significance testing for deviation from Hardy-Weinberg Equilibrium (HWE) test was conducted on the complete dataset using default settings. This data was not used for filtering of loci from either dataset since the collections don’t represent single populations and loci under divergent selection are expected to violate HWE. Neighbor-joining trees were generated using both the neutral and outlier datasets using Nei’s genetic distance method.

The R package ‘poppr’ was used to conduct Analysis of Molecular Variance (AMOVA) on both datasets^37^. The GenePop R package was also used for significance testing of pairwise F_ST_ to determine the genetic differences between collection sites using the neutral and outlier datasets using default settings with the samples grouped by collection site. Isolation by Distance analysis using the outlier dataset was done using the ‘adegenet’ R package using a pairwise distance matrix for all collection sites. Significance testing for isolation by distance was done by Mantel test using an Edward’s genetic distance matrix and a physical distance matrix between the collection sites with 9,999 iterations (also using the ‘adegenet’ package).

Principal Component Analysis (PCA) was done using the adegenet R package on both neutral dataset (14,815 SNPs) and outlier dataset (79 SNPs). The Bayesian clustering method implemented in the STRUCTURE software v. 2.3.4^38^ was used to genetically assign individuals to clusters. Simulations were run for 100,000 steps following a burn-in period of 100,000 steps, considering values of K (number of clusters) from one to 15, with 10 replications for each value of K. The analysis was performed using admixture, correlated allele frequencies and no prior information regarding sampling location or morphological species. For each individual the program identifies the fraction of the genome that belongs to each one of the clusters. The rate of change in the log likelihood between successive K values^39^ was also estimated. The calculations were performed with STRUCTURE HARVESTER^40^. The clusters of the estimated population structure were visualized by using CLUMPAK^41^. Clustering analysis using Discriminate Analysis of Principal Components (DAPC) using the outlier dataset was also done using ‘adegenet’ in order to reveal possible genetic clustering among samples without grouping by collection sites.

### Data accessibility

Our raw data with SRA accession number PRJNA503852 are available online in the NCBI sequence read archive (https://www.ncbi.nlm.nih.gov/sra/PRJNA503852, data publicly released on 31st December 2018).

## Result

### NextRAD Sequencing, Annotation and GO Categorization of Neutral and Adaptive Loci

On average, 2 million reads of 150 bp per individual were generated from the 180 nextRAD-genotyped *T. ilisha*. Out of the 180 individual fish, 13 individual sequences with greater than 20% missing genotypes were removed from the dataset, while the remaining 167 samples were used for all downstream analyses. Out of a total of 46,307 loci within a catalog of 92,971 consensus sequences, only 31,276 loci were remained when indels and SNP sites with less than 5% minor allele frequency have been removed from the original dataset. Further filtering for completeness of data at or above 80% for both samples and loci left a final set of 14,894 loci for analysis. As no reference genome was available for this species during the analyses; a *de novo* assembly was constructed using a custom script generated by SNPsaurus. We conducted homolog search of each contig in the assembly in the RefSeq database, and discovered that only 1.95% (1,814 of 92,971) of the contigs returned matching coding regions.

**Figure 2:**
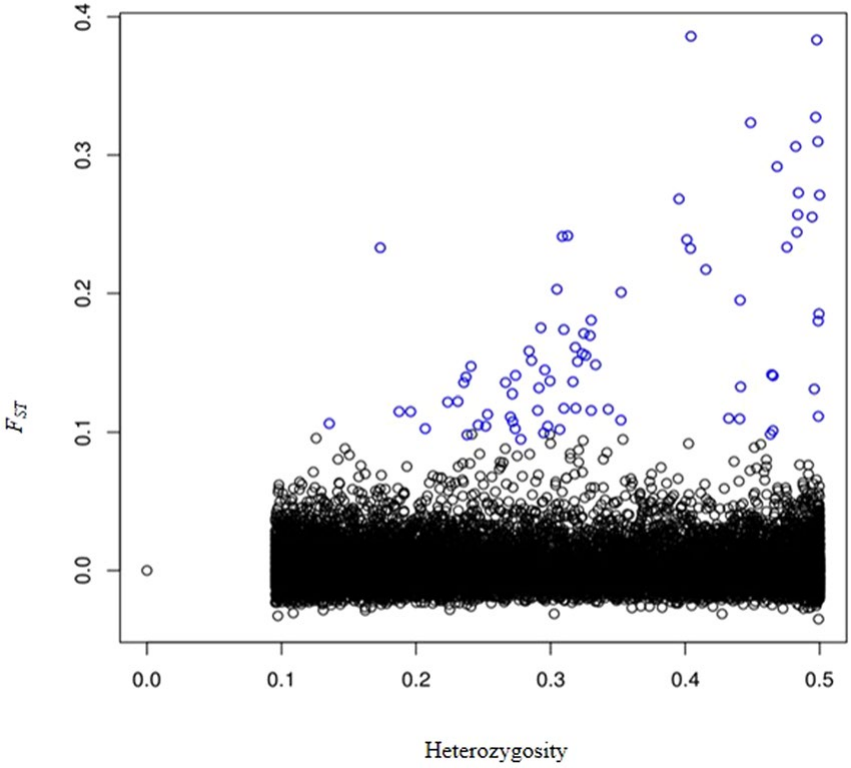
Result from the R Package OUTFLANK F_ST_ outlier analysis for 14,815 SNPs. Among these, 79 SNPs (blue open circles) are above a 0.995 probability and were considered as putatively adaptive loci under selection with highest F_ST_ values. The remaining SNPs (black open circles) were considered neutral.

Hardy-Weinberg (HWE) tests of the 14,894 loci revealed that an approximately 15% of these loci were significantly deviated from the HWE expectations. Some of the loci with a significant heterozygote deficit may be linked to adaptive variations and population structuring between sampling sites. Outlier analysis run in R Package OUTFLANK identified 79 putatively adaptive SNPs as being F_ST_ outliers and candidates for positive selection loci (P > 0.995) (Fig. 2). Neutral loci were determined using a conservative threshold range of probabilities between 0.10 and 0.90. There were 14,815 putatively neutral SNPs that fell within these probability levels.

**Figure 3:**
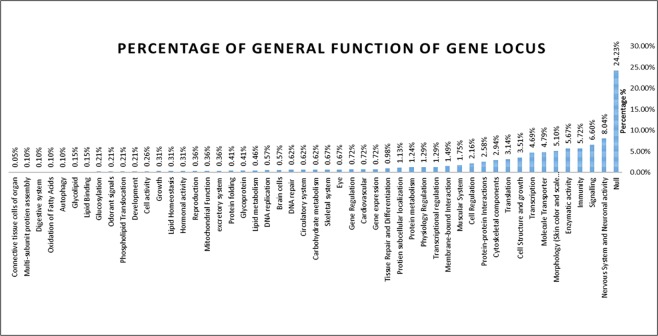
Gene ontology classification of 1,814 assembled contigs of *Tenualosa ilisha*

From the 14,894 loci being analyzed, only 1,814 loci (0.06%) were significantly matched to known genes in the public Nr databases and has been categorized into 51 groups with general functions of biological processes, molecular functions, cellular components and systemic functions (Fig. 3). 27 groups have shown a significant percentage from 1% up to 24%, while the remaining 24 groups remain less than 1%. Out of 79 identified putatively adaptive loci, only 14 loci were observed to be in the coding region and their gene functions are depicted in Table 2. Among the 14 putatively adaptive SNP loci, three loci (19201_25, 12546_217 and 74884_159) encodes the genes mostly involved in mitochondrial function and are mainly responsible for energy metabolism (Table 2). Other three loci (82109_13, 52941_15 and 44093_43) were found to be involved in neuronal activity important for neural communication and responsible to control a range of behavioral phenotypes. Other loci encode the gene mainly involved in transcription (9754_9 and 51360_15), reproduction (8853_12), cell growth (74886_11) and different signaling pathways (5615_159, 35109_9 and 44450_13).

**Table 2:** Summary of gene functions and annotation based on reference genome for 14 putatively adaptive loci that differentiates the *Tenualosa ilisha* populations in Bangladesh.

Locus	GenBank Accession Number	Gene	Species	Gene Function
9754_9	XM_012816864.1	KAT8 regulatory NSL complex subunit	*Clupea harengus*	Transcription: histone acetylation
8853_12	XM_012824429.1	leucine-rich repeats and guanylate	*Clupea harengus*	Reproduction: spermatogenesis and cell differentiation
82109_13	XM_012814935.1	Dmx-like 2 (dmxl2), mRNA	*Clupea harengus*	Neuronal activity: a key controller of neuronal and endocrine homeostatic processes
74886_11	XM_012821414.1	FYN proto-oncogene, Src family tyrosine	*Clupea harengus*	Cell growth
74884_159	AP011610.1	mitochondrial DNA, complete genome	*Tenualosa ilisha*	Mitochondrial function
5615_159	XM_010865640.1	MAP/microtubule affinity-regulating kinase	*Esox lucius*	Signalling: cytoskeletal signalling, glucose or energy metabolism
52941_15	XM_006792985.1	neurexin-3b-like	*Neolamprologus brichardi*	Neuronal cell surface protein associated with a range of behavioural phenotypes
51360_15	XM_012823915.1	cleavage and polyadenylation specific	*Clupea harengus*	Transcription: plays a central role in 3-prime processing of pre-mRNAs
44450_13	XM_017701045.1	inositol-trisphosphate 3-kinase	*Pygocentrus nattereri*	Protein-protein interactions: involved in inositol metabolic process, signal transduction and cytoskeleton organization
44093_43	XM_016260991.1	glutamate receptor ionotropic	*Sinocyclocheilus grahami*	Neuronal activity: important for neural communication, memory formation, learning, and regulation
35109_9	XM_013137472.1	ryanodine receptor 3-like	*Esox lucius*	Signalling**:** cellular mediator of Ca^2+^ release channels and thereby plays a role in triggering muscle contraction
19201_25	AP011611.1	mitochondrial DNA, complete genome	*Tenualosa ilisha*	Mitochondrial function
16116_52	XM_012835890.1	C-type lection lectoxin-Enh3-like	*Clupea harengus*	Mannose-binding lectin and agglutinates a variety of animal cells
12546_217	FJ582830.1	cytochrome oxidase subunit 1	*Amphiprion sebae*	Mitochondrial function: involved in energy metabolism and aerobic respiration

### Population structure analysis based on phylogenetic analysis

**Figure 4:**
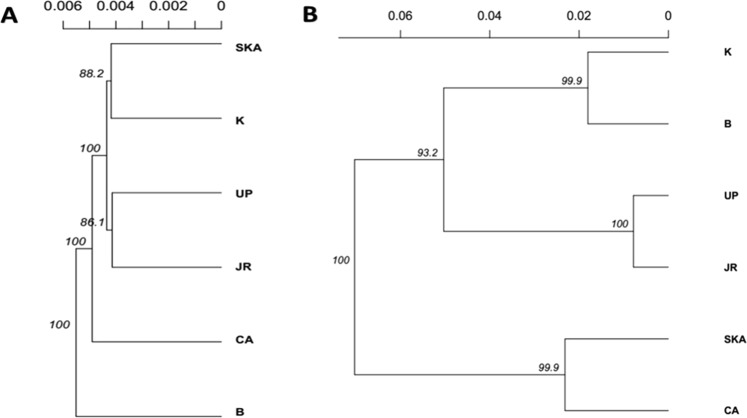
Neighbor-joining trees based on Nei’s genetic distances using the following sets of loci: (**A**) putatively neutral panel of SNPs, and (**B**) the putatively adaptive panel of SNPs. Branch nodes are denoted as the percentage of bootstrap support that was generated with 1,000 replicates. Collection codes correspond to those listed in Table 1.

Neighbor-joining (NJ) analysis of 14,815 putatively neutral loci (Fig. 4a) revealed weak structure, and showed that the populations were not delineated strictly into their respective units as observed in the outlier loci dataset. Two main groupings were formed: (i) (Surma-Kushiara and Kuakata = 88.2% bootstrap support), (ii) (Upper Padma and Jamuna = 86.1% bootstrap support), and the remainder of the collections branched individually (Chandpur and Bhola). The phylogenetic analyses of the 79 putatively adaptive loci showed the following three main groupings of collections: Marine and Estuary (Kuakata and Bhola = 99.9% bootstrap support), North-Western Riverine (Upper Padma and Jamuna = 100% bootstrap support), and North-Eastern Riverine (Surma-Kushiara and Chandpur = 99.9% bootstrap support) (Fig. 4b). Overall, the terminal branch lengths were longer in the NJ tree based on adaptive loci when compared to the NJ tree generated from neutral loci dataset, suggesting substantial genetic variations among populations in their respective clusters (Fig. 4).

### Demographic inferences from AMOVA, F_ST_ statistics, PCA and DAPC analysis

For the AMOVA analysis, 27 loci were dropped from the outlier dataset and 5,593 loci were dropped from the neutral dataset (approximately 1/3 of the loci in each set) (Table 3, Fig. S1). In both cases, variation between populations was less than observed variation among samples within collection sites. Consistent with the NJ clustering analysis, AMOVA analysis of the outlier dataset also showed best support for the population differentiation, yielded an overall F_ST_ of 0.1826 (P = 0.001). The largest component of genetic variability was explained at the individual level (63.8%). The portion of variation captured among the six populations was 18.6% (P = 0.0001), while the remaining variation among individuals within population was 17.6% (P = 0.0001). An AMOVA analysis based on these neutral loci dataset yielded a mean F_ST_ of 0.0029 (P = 0.001). The highest genetic variation was observed among individuals (86.8%), with little genetic variation among individuals within population (12.9%, P = 0.0001) and even significantly smaller variation between populations (0.3%, P = 0.0001).

**Table 3:** Analysis of Molecular Variance (AMOVA) among 6 sampling locations of *Tenualosa ilisha* distributed in aquatic ecosystem of Bangladesh.

Source of variation	Sigma	% of variation	P-value
***For neutral SNPs loci***			
Between population	7.523	0.2901	0.0001
Among samples within population	334.749	12.911	0.0001
Among samples	2250.472	86.798	—
***For adaptive SNPs loci***			
Between population	3.474	18.607	0.0001
Among samples within population	3.294	17.642	0.0001
Among samples	11.90413	63.751	—

**Figure 5:**
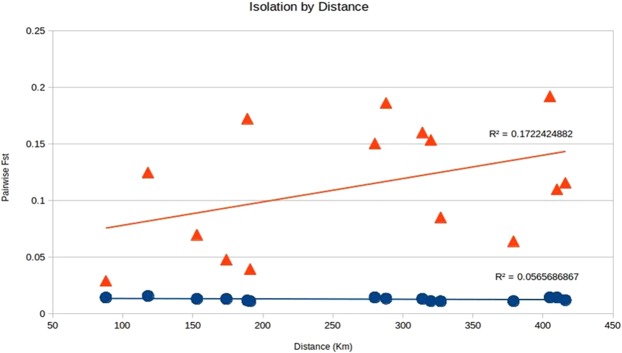
Correlation of pairwise *F*_*ST*_ value with pairwise geographical distances among *Tenualosa ilisha* collection. The red triangles represent the putatively adaptive loci *F*_*ST*_ and the blue circles are the neutral loci *F*_*ST*_. The Mantel test showed simulated p-value of 0.0134 based on 9,999 replicates indicating significance effect.

The pairwise F_ST_ values for the adaptive loci dataset were markedly higher than the F_ST_ values for the neutral loci dataset, supporting genetic distinctiveness between the Hilsa shad populations (Table 4). For the 14,815 putatively neutral loci, pairwise F_ST_ values ranged from 0.0109 to 0.0156 and averaged 0.0132 (Table 1). The pairwise F_ST_ analysis for 79 putatively adaptive loci showed an above average range across all comparisons ranging from 0.0289 to 0.1919 and averaged at 0.1100. Individuals from Estuary (B) and Jamuna River (JR) were observed to have the highest pairwise F_ST_ values (F_ST_ = 0.19192, P = 0.001; Table 2). However, all of the pairwise F_ST_ values for both neutral and adaptive panels of SNP loci showed significant difference between them (Table 2). We also investigated the correlation of population differentiation (F_ST_) and geographic distance. Isolation-by-Distance (IBD) analysis of neutral loci generated a regression slope of 0.0565, and the Mantel test was insignificant (P > 0.05). We found a statistically significant relationship between increasing degree of geographic separation and increasingly large F_ST_ for the putatively adaptive loci, in which the IBD slope was significantly higher at R^2^ = 0.1722 (P = 0.0134) (Fig. 5).

**Table 4:** Pairwise F_ST_ values for the putatively neutral SNPs (above diagonal) and putatively adaptive SNPs (below diagonal) in Hilsa shad.

	K (SS)	B (ME)	CA (MR)	SKA	UP	JR
K (SS)	—	0.01422 (0.001)	0.01309 (0.001)	0.01094 (0.003)	0.01106 (0.002)	0.01194 (0.001)
B1 (ME)	0.02888 (0.019)	—	0.01561 (0.001)	0.01437 (0.001)	0.01432 (0.001)	0.01421 (0.001)
CA (MR)	0.06975 (0.002)	0.12462 (0.001)	—	0.01296 (0.001)	0.01312 (0.001)	0.01309 (0.001)
SKA	0.08507 (0.001)	0.15021 (0.001)	0.04765 (0.016)	—	0.01113 (0.002)	0.01173 (0.001)
UP	0.06401 (0.001)	0.10978 (0.001)	0.16010 (0.001)	0.15341 (0.001)	—	0.01100 (0.002)
JR	0.11544 (0.001)	0.19192 (0.001)	0.18607 (0.001)	0.17230 (0.001)	0.03931 (0.006)	—

Collection codes correspond to those listed in Table 1. P value are shown within the parenthesis for each pairwise F_ST_ value.

We have also conducted a pairwise comparison of F_ST_ values based on 79 putatively adaptive loci between adult and juveniles for five sampling sites except individuals from Meghna estuary in Bhola (Table 5). Notably, juveniles from three sampling sites (Surma-Kushiara, Upper Padma and Jamuna) were observed to show extremely weak genetic differentiation with their respective adults with non-significant zero F_ST_ values. Interestingly, the genetic variation between adults from Meghna River in Chandpur (CA-A) and juveniles from Surma-Kushiara (SKA-J) was also found to be zero (F_ST_ values = 0.0000). However, juveniles from these three sites showed a significant higher genetic differentiation with the adults of the other collection sites. In contrast, small, but significant distinction was seen between adults and juveniles in Meghna River at Chandpur (F_ST_ values = 0.06296, P = 0.003) and sea at Kuakata (F_ST_ values = 0.07787, P = 0.002).

**Table 5:** Pairwise F_ST_ values based on 79 putatively adaptive loci between adult and juveniles for five sampling sites in Bangladesh.

	K-J	CA-J	SKA-J	UP-J	JR-J
K-A	0.07787 (0.002)	0.21494 (0.001)	0.11007 (0.001)	0.14356 (0.001)	0.19765 (0.001)
CA-A	0.05743 (0.004)	0.06296 (0.003)	0.00000 (0.594)	0.22993 (0.001)	0.26771 (0.001)
SKA-A	0.02957 (0.028)	0.15486 (0.001)	0.00000 (0.582)	0.07906 (0.002)	0.23532 (0.001)
UP-A	0.03346 (0.016)	0.13463 (0.001)	0.12698 (0.001)	0.00000 (0.592)	0.09103 (0.001)
JR-A	0.11087 (0.001)	0.13409 (0.001)	0.32294 (0.001)	0.02339 (0.032)	0.00000 (0.564)

Collection codes correspond to those listed in Table 1. “J” indicates juvenile Hilsa shad and “A” indicates adult Hilsa shad. P value are shown within the parenthesis for each pairwise F_ST_ value.

**Figure 6:**
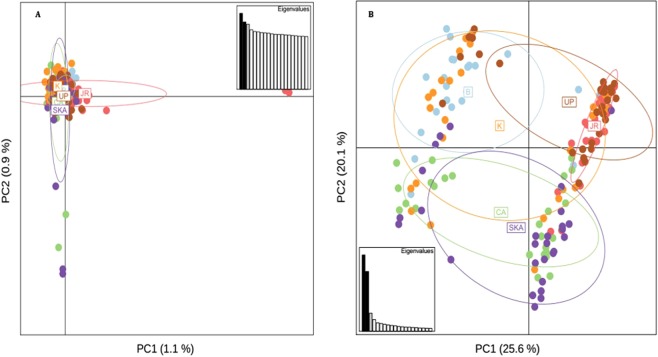
Scatterplots showing the Principal Components Analysis (PCA) for: (**A**) the putatively neutral panel of SNP loci and (**B**) the putatively adaptive panels of SNP loci. Collection codes correspond to those listed in Table 1. PCAs determined optimal clustering of three groupings (1) marine/brackish represented by two closest relatives at Kuakata sea (K) and the Meghna Estuary, Bhola (B), (2) muddy freshwater represented by two closest relatives at the Upper Padma (UP) and the Jamuna (JR), and (3) clear freshwater types represented by the Meghna River (CA) and its upstream tributaries, Surma-Kushiara (SKA). Both the neutral and outlier datasets were highly significant for population differentiation via GenePop test.

**Figure 7:**
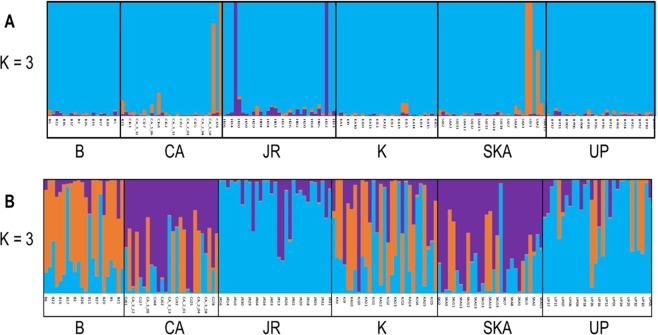
Bayesian STRUCTURE bar plot based on the (**A**) the putatively 14,815 neutral SNP loci and (**B**) the putatively 79 adaptive SNP loci of 180 *Tenualosa ilisha* individuals. Black lines separate individuals of different populations. Each vertical line represents an individual. The colors represent the proportion of inferred ancestry from K ancestral populations. Based on the delta K statistic, the best supported number of a posteriori genetic clusters was K = 3 for the standard admixture model (Table S1).

**Figure 8:**
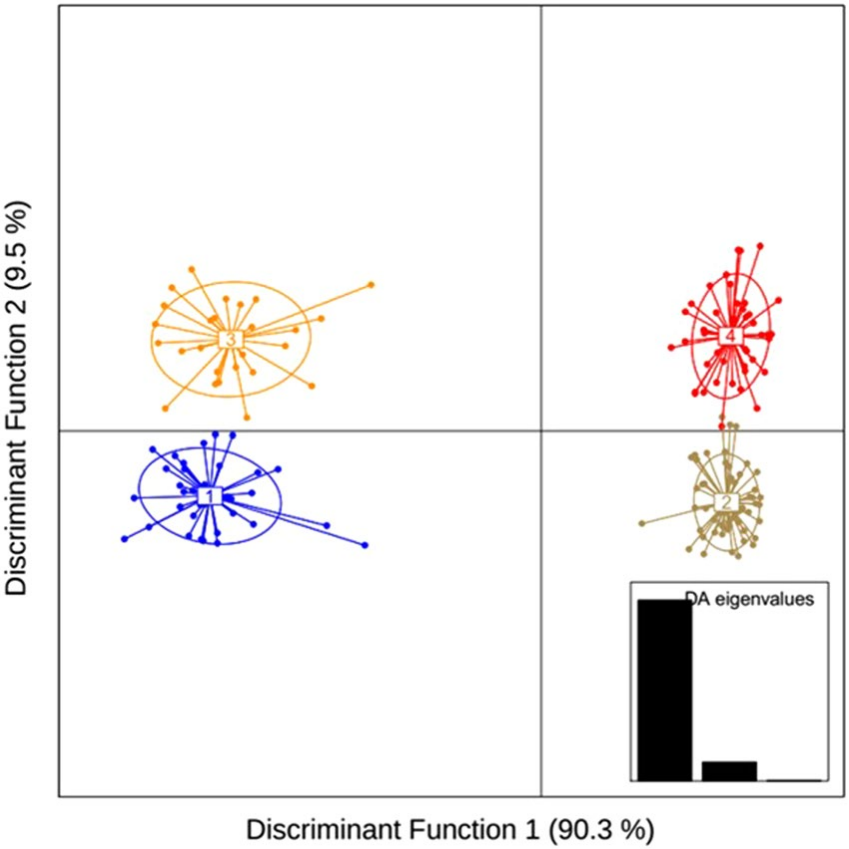
Scatterplots showing Discriminant Analysis of Principal Components (DAPC) of genetic differentiation among the 180 *Tenualosa ilisha* for the 79 putatively adaptive panels of SNPs loci. Ovals are the inertial ellipse, dot represent individual genotypes and the line extends to centroids of each population. DAPC grouped the collection sites of *T. ilisha* into four major clusters.

**Figure 9:**
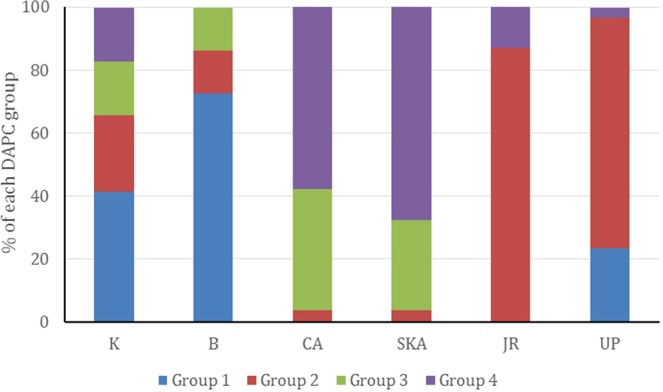
Bar graph showing *Tenualosa ilisha* collection sites-wise DAPC grouping. Collection codes in X-axis correspond to those listed in Table 1.

Principal component analyses on both outlier and neutral SNPs revealed an overall different population structure (Fig. 6). PCA of the neutral SNPs dataset supported a single homogenous population, consistent with the low genetic differentiation as indicated by pairwise F_ST_ values. On the other hand, PCA based on the adaptive SNPs showed three major clusters (Marine and Estuary, North-Western Riverine and North-Eastern Riverine) without clear separation of the populations according to the collection sites, which is found to be consistent with the NJ clustering, AMOVA analysis and F_ST_ values. Similarly, STRUCTURE analysis (Fig. 7) have also shown similar clustering pattern of the six Hilsa shad populations into three main genetic clusters based on the delta K statistic (K = 3). In contrast with the PCA, NJ clustering and STRUCTURE analyses, our DAPC analysis based on the adaptive SNPs dataset identified four distinct partitioning of populations, although the population structure was not explainable by the collection groups (Fig. 8). However, based on the DAPC output, we further unravel the assignment of individuals to their respective clusters (Fig. 9). The distinctive fourth clusters observed in non-model-based spatial analysis (DAPC) were embedded in a major cluster of the model-based analysis (NJ tree), as the North-Eastern Riverine (Surma-Kushiara and Chandpur). The DAPC assignment analysis showed that Surma-Kushiara and Chandpur was further divided into two separate clusters.

## Discussion

The use of genomic approaches to study population structuring and species demographics has become increasingly common with high throughput sequencing platform, supplementing findings from previous studies that have used traditional genetic markers such as allozyme, mitochondrial DNA and microsatellites^42^. We used nextRAD sequencing to generate a panel of genome-wide SNPs to examine fine-scale population structuring in *T. ilisha* in their native distribution regions throughout Bangladesh.

Our AMOVA and F_ST_ analyses of 79 putatively adaptive loci indicate substantial levels of genome-wide differentiation among the six populations. The neighbor-joining tree (NJ) and spatial analyses (PCA, STRUCTURE and DAPC) generated by small panel of highly differentiated loci (79 putatively adaptive loci) provided strong evidence for three major genetically separated groups of Hilsa shad in Bangladesh: Marine and Estuary (Kuakata and Bhola), Western Riverine (Upper Padma and Jamuna), and Eastern Riverine (Surma-Kushiara and Meghna). This is consistent with the findings of^19^ based on RAPD markers, of^17^ based on RFLP of mitochondrial DNA, and of^18^ based on PCR-RFLP of mitochondrial D-loop region. These studies came to a consensus that Hilsa shad populations were genetically diverged by three different environments. This broad structuring in three independent groups in turn mirrors the spatial structuring which is likely maintained by environmental differences such as turbidity and salinity levels in the marine or brackish, muddy/turbid freshwater (Western Riverine) and clear freshwater (Eastern Riverine) habitat^43^. Fishes of Western Riverine habitats are of bright silvery in color with thicker structure that could be the influence of the whitish silty turbid water, while fishes of Eastern Riverine are thinner, a bit darker and elongated that could be the influence of dark colored water^43^. Likewise, biometrical comparisons have also delineated Hilsa shad into three stocks according to different ecotypes^44–46^. Our spatial analysis and low F_ST_ value (0.02888) did reveal that population of Kuakata and Bhola appear to be intermediate which may be indicative of gene flow and admixture events, as both locations seems to be adjacently located to each other in the further southern coastal regions (Fig. 1). In fact, there was evidence stating the ability of *T. ilisha*, to spawn in the estuary, although no report has indicated any marine spawning in Bangladesh^47^. Therefore, fishes from Kuakata may have migrated to Bhola to spawn, but not to any other rivers, as significant differentiations between Kuakata and other river populations have been observed. Two clusters which were found within the North-Eastern and Eastern Riverine group (Surma-Kushiara and Chandpur) may likely represent two separate breeding grounds within the same riverine system, which is also congruent with previous studies^48^. Besides, the presence of population structuring in Hilsa shad was also reported by studies conducted using allozymes markers^14,49,50^ and RAPD analysis^20,21^.

On the other hand, there was no evidence of genetic differentiation between all studied populations based on multiple analyses of 14,815 neutral loci dataset in this study. Our results were consistent with findings from other studies using various markers and techniques such as allozymes^15,16,51^, otolith microchemistry and morphometrics^8^, mitochondrial cytochrome b^21,23^ and mitochondrial control region^52^. The lack of detectable population structural differences among the Hilsa shad in their distributed regions in Bangladesh suggests that there was extensive movement and mixing of Hilsa shad throughout Bangladesh for breeding purpose, given its anadromous nature^53^. Indeed, reproductive isolation may be likely impeded by the influx of large quantities of freshwater during monsoon, seasonal water circulation and continuous migration of Hilsa shad^54^. Therefore, these populations should be considered as belonging to a single gene pool and managed as a panmictic population^8^.

However, the lack of population genetic structure does not preclude a genetic basis for the presence of genetic differentiation as postulated by the presence of putatively adaptive loci, which have clearly separated the populations. It is highly possible that the neutral loci and previously used methods were unable to detect the smaller-scale genetic difference between the three ecotypes identified by putatively adaptive loci. Moreover, we also hypothesized that the lower water discharge from the upstream river flow from Farakka barrage (in the West Bengal, India) with constant heavy siltation may have disrupted the migration routes of the anadromous Hilsa shad ascending tributaries in the Western Riverine habitats^11^. Previous meristic and morphometric studies have also revealed some morphological differences among the Hilsa stocks from different environments^55^. Owing to the distinctive differences observed in the body height, the Hilsa stocks were divided into Western Riverine’s ‘Broad type’ and Eastern Riverine’s ‘Slender type’^56–58^. The different seasonal spawning between these two morphotypes: the “broad type” during monsoon and “slender type” during winter, and each with different fecundity level, may have also contributed to the limited natal and breeding dispersal, and thus population divergence of Hilsa shad^59^.

We identified that some genes of the 14 putatively adaptive loci codes for multiple transcript variants encoding different isoforms, where genetic variation at this locus has been associated and may result with a range of behavioral phenotypes^60^. Isoforms are messenger RNAs resulted from transcription of the same gene locus but at a different transcription start sites (TSSs) or untranslated regions (UTRs), which results in alteration of gene functions^61^. This molecular complexity may lead to phenotypic differences and genetic variation between individuals at cellular level and thus deliberating the potential of population divergence in Hilsa shad^62,63^. From the match analysis of putatively adaptive loci, gene from the 51,360_15 locus encodes cleavage and polyadenylation specificity factor (CPSF), which is involved in the cleavage of the 3’ signaling region from a newly synthesized pre-messenger RNA (pre-mRNA) molecule in the process of gene transcription (Table 2). On a similar study of population genomics, through transcriptome analysis it was found that intron retention and alternative polyadenylation is largely independent along the transcript, leading to thousands of novel isoforms. With that, the study was concluded by proposing that the independent combination of alternative RNA processing events has contributed to complex isoform evolution in the organism, which provides a new foundation for the study of phenotypic difference among the population^64^, where similar mechanism can be expected in the phenotypic variations in the Hilsa shad population species in Bangladesh.

Three genes from the match value (19201_25, 12546_217 and 74884_159) associates with the mitochondrial DNA, function and mitochondrial cytochrome oxidase subunit 1 (Table 2). Various studies have shown the relation of mitochondrial DNA and diversity in a population and the study of this analysis has demonstrated large values of nucleotide and a set of genetic determinants located on a single chromosome called haplotype. The presence of shared haplotypes in most populations is either the restricted gene flow among local populations or the predominance of ancestral haplotypes^65^. Although mitochondrial COI gene have been widely used for species identification studies, it is also used for estimation of inter- and intra-specific diversity in many organisms^65,66^. A significant weightage of greater than 5% were morphology (skin and scale pattern), enzymatic activity, immunity, signaling, nervous system and neuronal activity (Fig. 3) may explain the variations between the Hilsa shad individuals of three different environments with a vast diverse of water parameters such as salinity and turbidity. Based on the functional annotations, the two main categories, the morphology (skin and scale pattern) and immunity may also play an important role in the distribution of Hilsa shad individuals based on their adaptability to external environment, prey and predation, immunity and disease resistance over a certain period.

While Hilsa shad populations were found to be genetically differentiated by three major groups due to geographical separation, juveniles and adults from each populations seems to be sharing the same genetic structure, except for individuals from Kuakata and Chandpur. The significant weak genetic structuring in three sampling locations (Surma-Kushiara, Upper Padma, and Jamuna) as revealed by zero F_ST_ values indicates that the juveniles are offspring of the existing adults and the adults may potentially return to their natal ground for spawning purposes. Given that the three rivers are geographically isolated, admixture events are not likely to happen. Contradicting genetic variation patterns were observed in the Surma-Kushiara River (SKA), in which adults from Meghna River in Chandpur (CA) were genetically similar to juveniles from Surma-Kushiara, which likely reflect the migratory behavior of some of the Chandpur River’s adults to the spawning ground in the upper stream of Surma-Kushiara River to breed. This finding was consistent with the major groupings in the above-mentioned analyses, in which individuals of Chandpur and Surma-Kushiara River were clustered as Eastern Riverine group. Unexpectedly, similar trend was not clearly depicted in both Chandpur (CA) and Kuakata (K), in which the adults and juveniles showed low genetic differentiation, but not sharing the same genetic structure as Surma-Kushiara, Upper Padma and Jamuna. This might be because that these two sites are the common routes of Hilsa shad to migrate from downstream to the geographically separated upstream rivers, thereby frequent admixture events may likely happen, and some adults may have been mixed during sampling activities. However, these observations may still allow us to conclude that Hilsa shad do return to their respective natal grounds for spawning, in which they are geographically separated. Fascinatingly, some putatively adaptive SNP loci (82109_13, 52941_15 and 44093_43) encode the genes, which are involved in neuronal function such as neural communication, memory formation and learning, in which lead to us to suggest that these genes might have played an important role in facilitating Hilsa shad to reach their natal ground in subsequent years for spawning.

### Conclusion and management implications

Although neutral loci did not reveal much genetic structural differences among the Hilsa shad populations, by using 79 adaptive loci, we were still able to observe genetic differences which are so distinctive that they warrant the division of Hilsa shad into three separate ecotypes in Bangladesh waters: (1) marine/brackish represented by two closest relatives at Kuakata and the Meghna Estuary, Bhola, (2) muddy freshwater represented by two closest relatives in the Upper Padma and the Jamuna, and (3) clear freshwater types represented by the Meghna River and its upstream tributaries, Surma-Kushiara. In a conservation management context, it is important to maintain the genetic diversity of Hilsa shad in each location, given that our results have suggested high levels of gene flow within populations but lower variation across most of their range. The most important phenomenon revealed from this study that has great management implications is the fact that Hilsa shad returns to their natal rivers for spawning. Hilsa fishers try to catch juveniles before they move to the sea for maturation, because of the fear that they might not be able to uphold their catch yield. This finding will help in motivating fishers not to catch the juveniles, as they will get the assurance of getting back the juvenile as adult in the respective rivers. Our findings also indicated the importance of incentive-based juvenile protection activities involving all stakeholders, including the fishers for each of the rivers where Hilsa shad spawn and juveniles spend their early life. We note that empirically testing these hypotheses will require further characterization of the remaining putatively adaptive loci based on a more complete whole-genome sequence and at a larger regional level constituting the native distribution of Hilsa shad. Ultimately, isolating mechanisms, environmental factors, and the temporal stability of these patterns in structuring these groups in Bangladesh will require further study to help fishery managers in formulating ecotypes oriented management strategies for sustainable fisheries of two million fishers who participate in the Hilsa shad fishery. We suggest that this innovative work provides a model that may be of value in other aquatic species, especially in anadromous fish species that intersect diverse ecotypes during their life-history stages.

## Supplementary information

Supplementary Dataset (STRUCTURE)

Supplementary Info
