# Supplementary material for: Fine-scale population structure and ecotypes of anadromous Hilsa shad (Tenualosa ilisha) across complex aquatic ecosystems revealed by NextRAD genotyping
Source: Sci Rep. 2019 Nov 5;9:16050. doi: 10.1038/s41598-019-52465-2 (PMC6831668; doi:10.1038/s41598-019-52465-2)
Supplement: Supplementary file 1 — Supplementary Dataset (STRUCTURE) [file 41598_2019_52465_MOESM1_ESM.pdf]

Fine-scale population structure and ecotypes of anadromous Hilsa shad (*Tenualosa ilisha*) across complex aquatic ecosystems revealed by NextRAD genotyping

Md Asaduzzaman[1], Md A. Wahab[2], Md J. Rahman[2], Md Nahiduzzzaman[2], Malcom W. Dickson[2], Yoji Igarashi[3], Shuichi Asakawa[3] and Li Lian Wong[4, 5]*

[1]Department of Marine Bioresource Science, Faculty of Fisheries, Chattogram Veterinary and Animal Sciences University, Khulshi 4225, Chattogram, Bangladesh

[2]WorldFish, Bangladesh and South Asia Office, Banani, Dhaka, 1213, Bangladesh

[3]Laboratory of Aquatic Molecular Biology and Biotechnology, Department of Aquatic Bioscience, The University of Tokyo, 1-1-1 Yayoi, Bukkyo-ku, Tokyo 113-8657, Japan

[4]Institute of Tropical Aquaculture, Universiti Malaysia Terengganu, 21030 Kuala Terengganu, Malaysia

[5]Institute of Marine Biotechnology, Universiti Malaysia Terengganu, 21030 Kuala Terengganu, Malaysia

[*]Corresponding author

Dr. Li Lian Wong

Phone: 609-668-3671

Fax: 609-668-3390

Email: lilian@umt.edu.my

CLUMPAK main pipeline - Job 1553223055 summary

Major modes for the uploaded data:

K=1

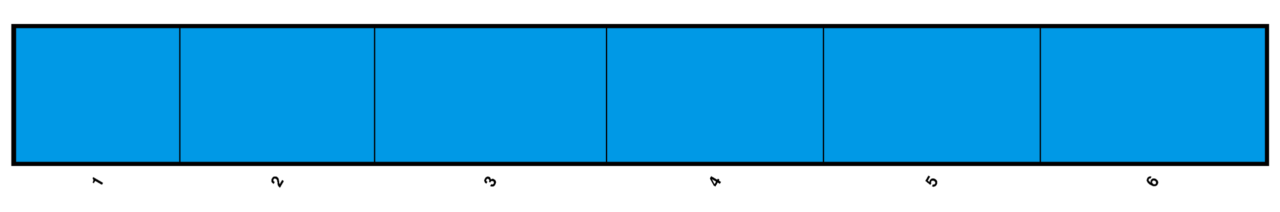

K=2

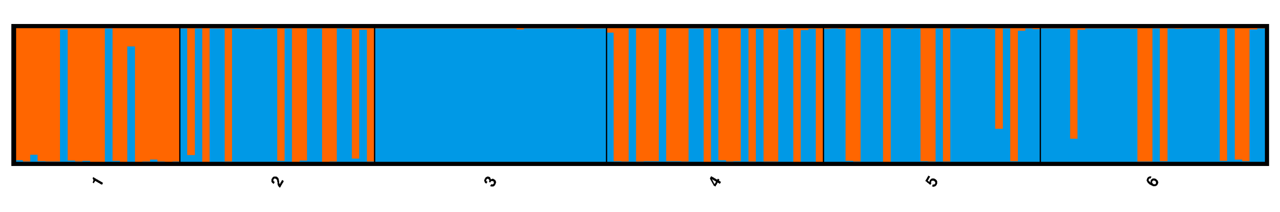

K=3

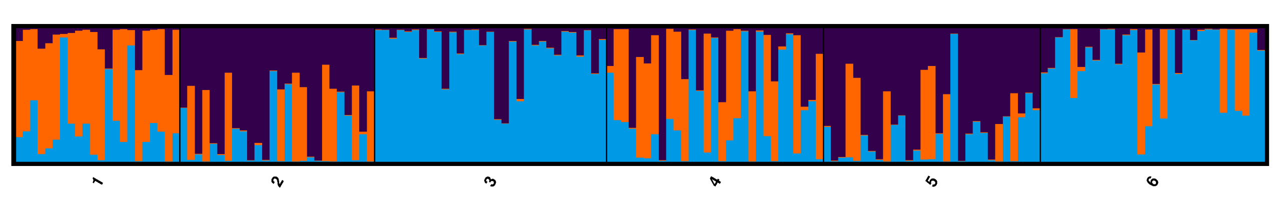

K=4

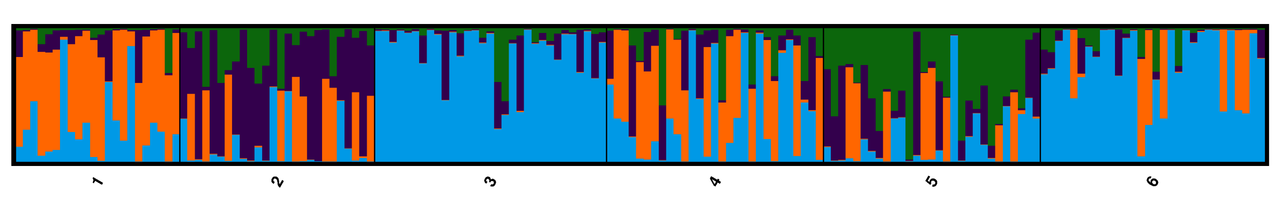

K=5

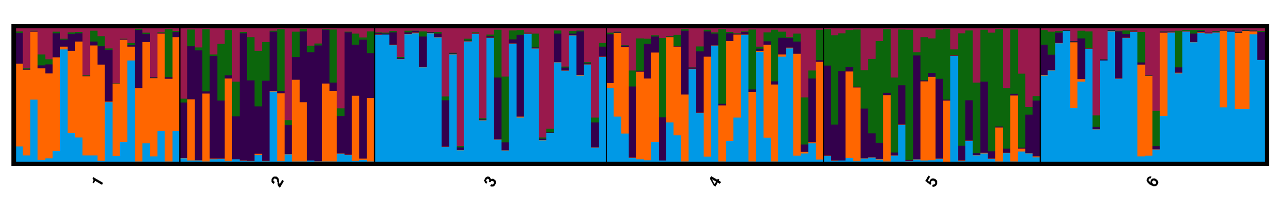

K=6

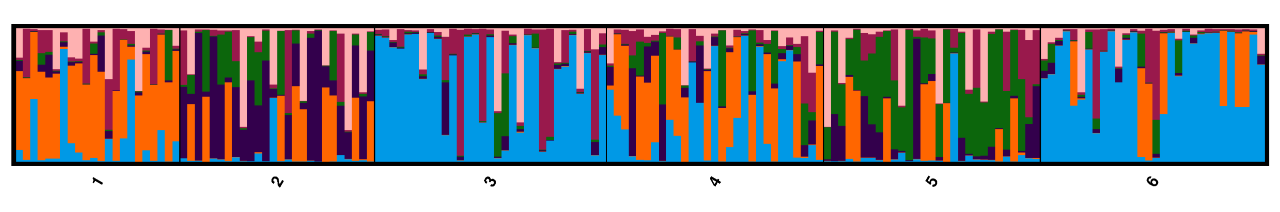

K=7

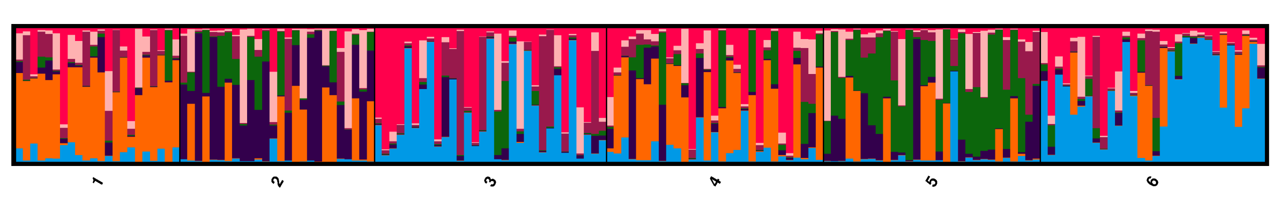

K=8

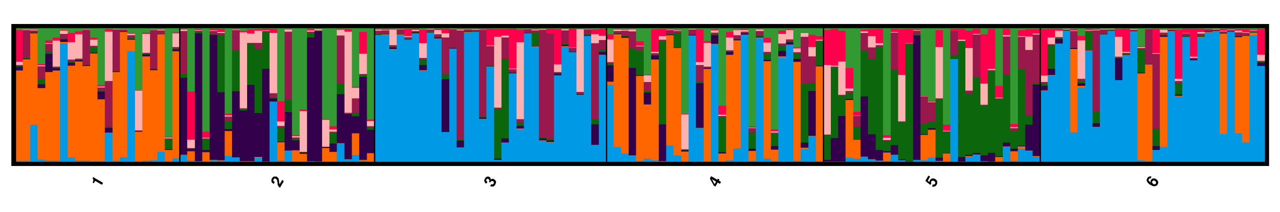

K=9

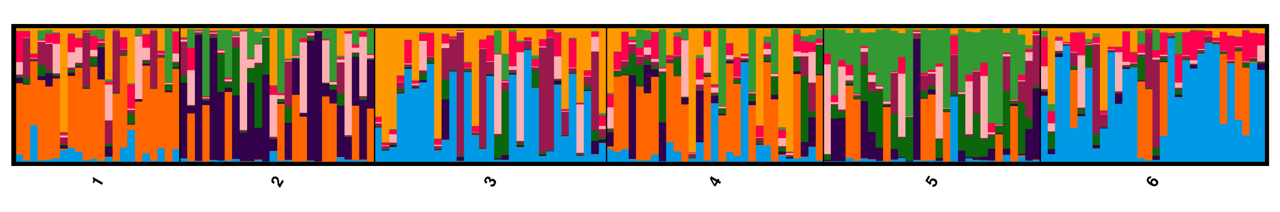

K=10

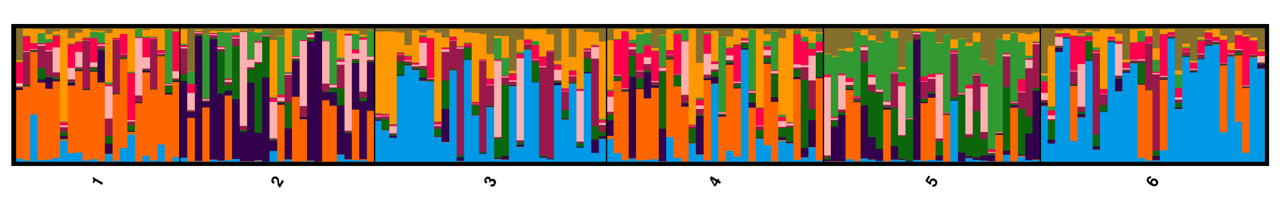

K=11

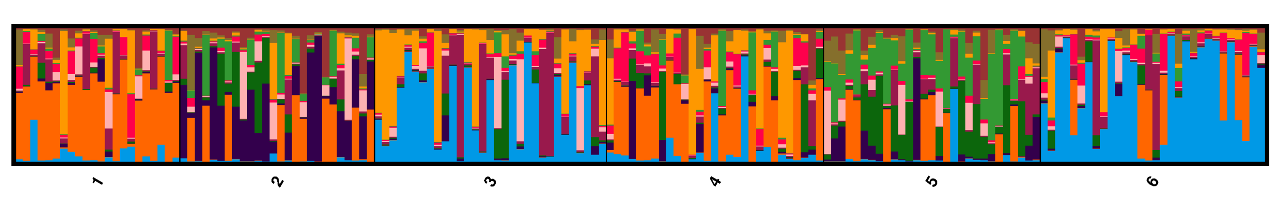

K=12

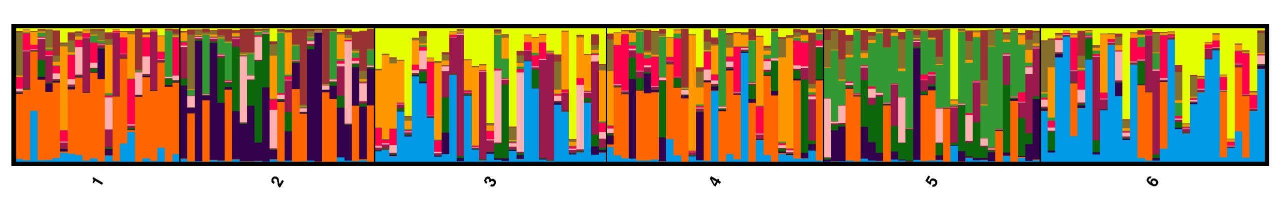

K=13

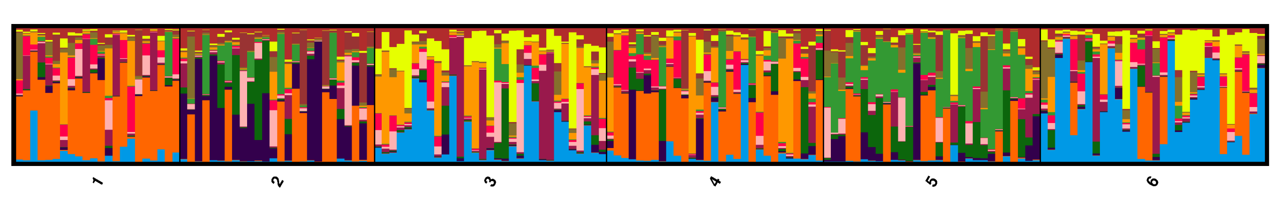

K=14

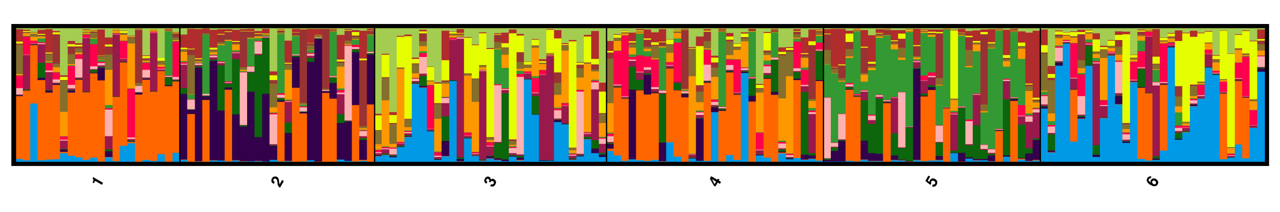

K=15

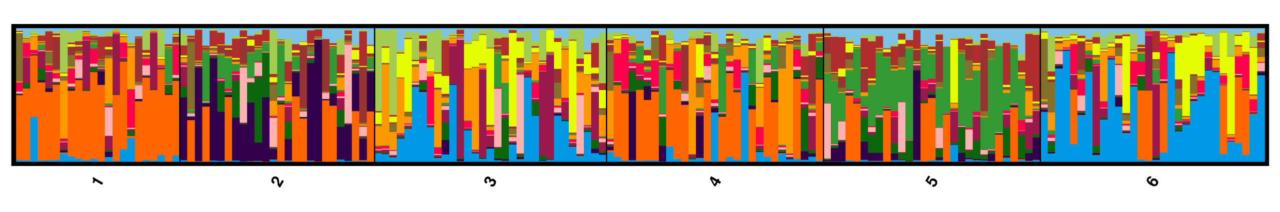

Minor modes for the uploaded data:

K=5 MinorCluster1

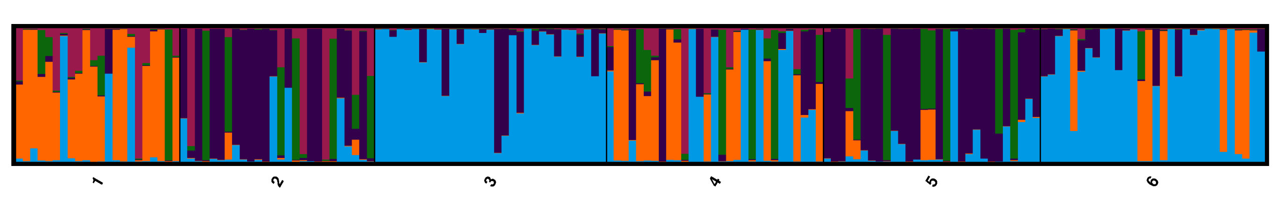

K=6 MinorCluster1

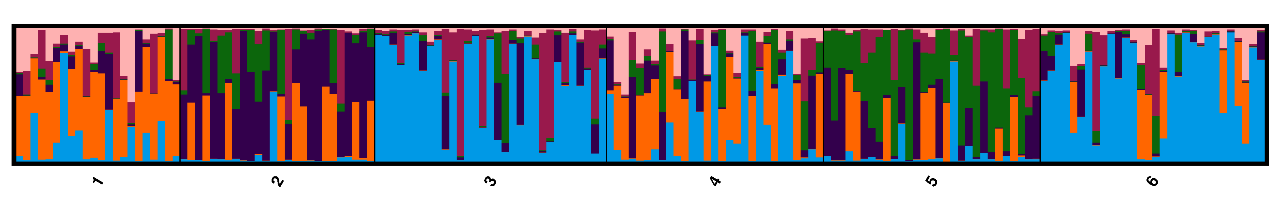

K=6 MinorCluster2

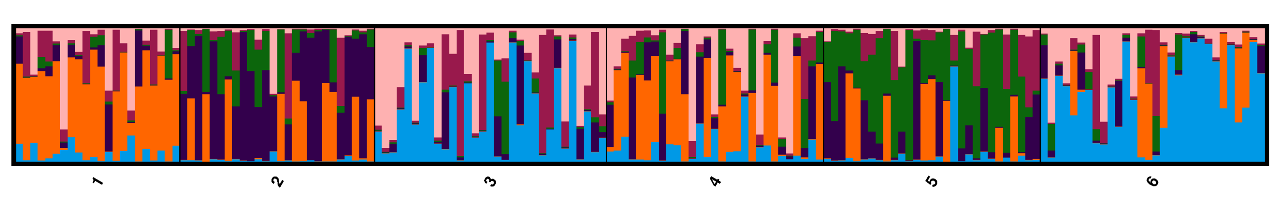

K=7 MinorCluster1

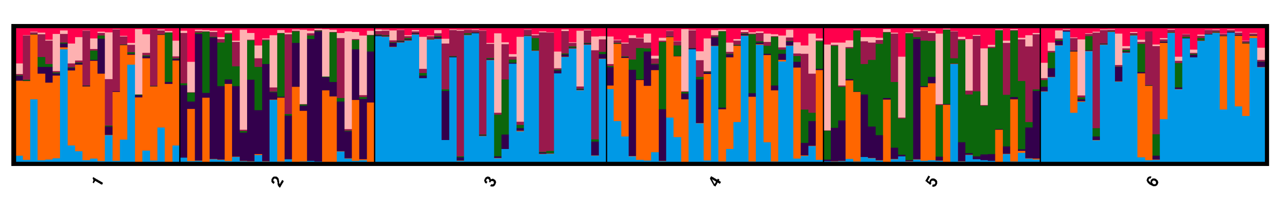

K=8 MinorCluster1

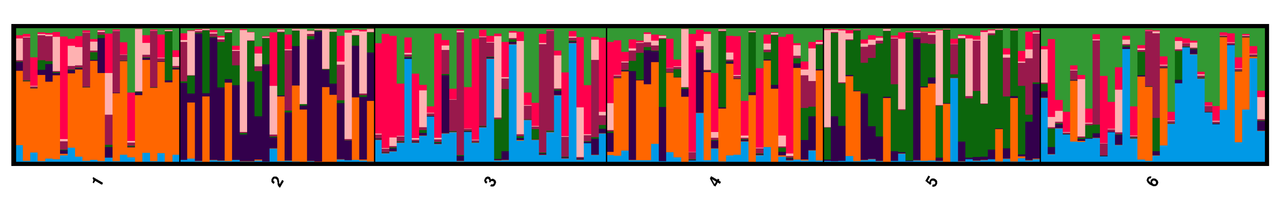

K=9 MinorCluster1

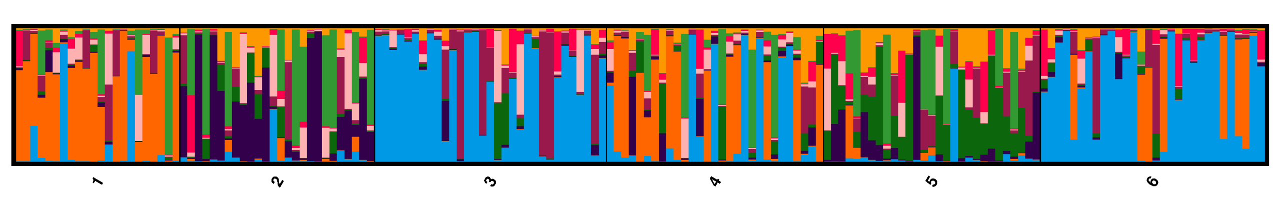

K=9 MinorCluster2

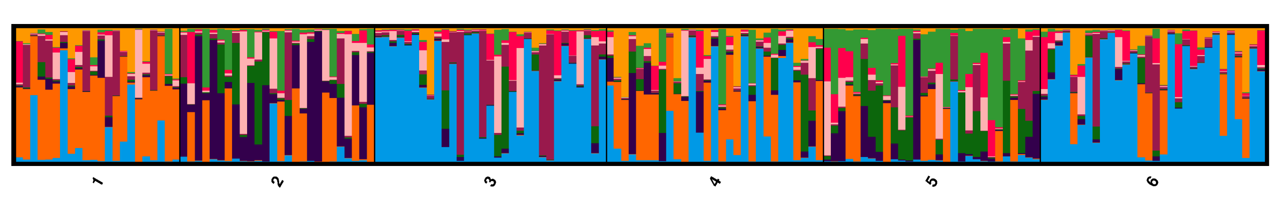

K=10 MinorCluster1

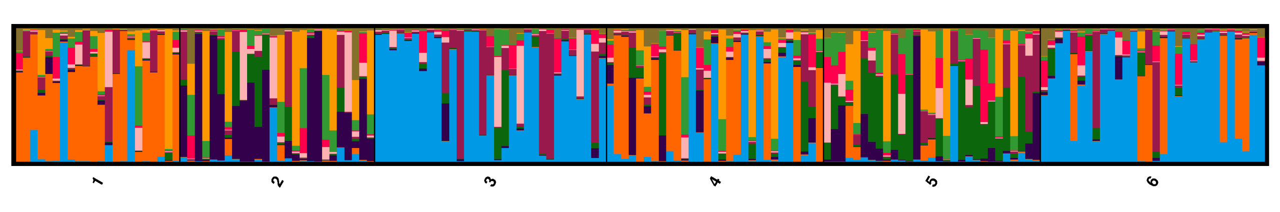

K=11 MinorCluster1

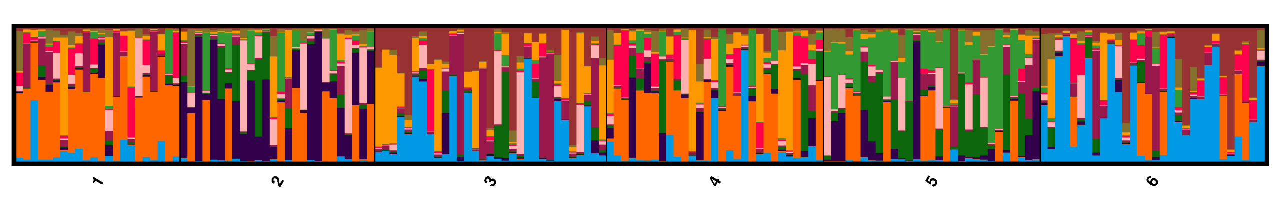

K=11 MinorCluster2

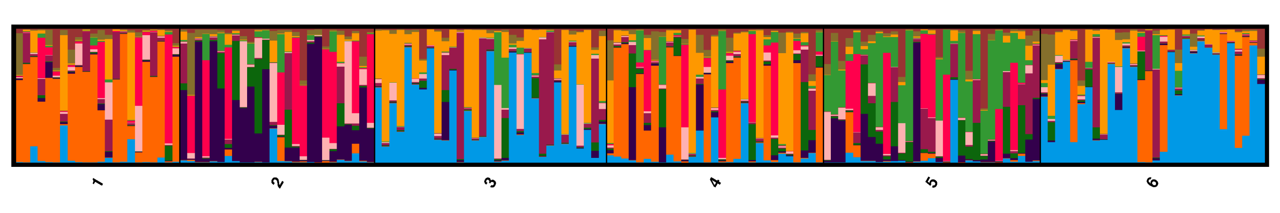

K=12 MinorCluster1

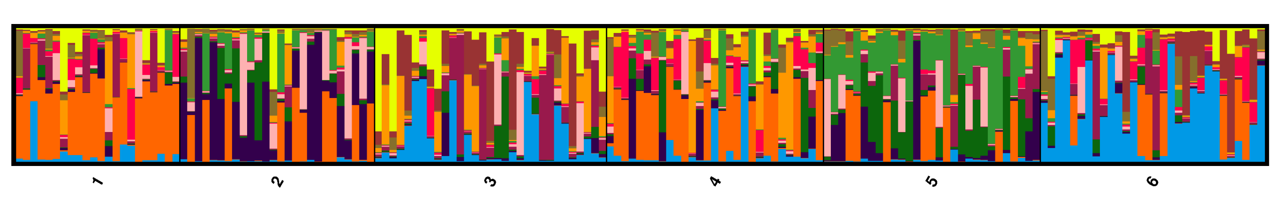

K=12 MinorCluster2

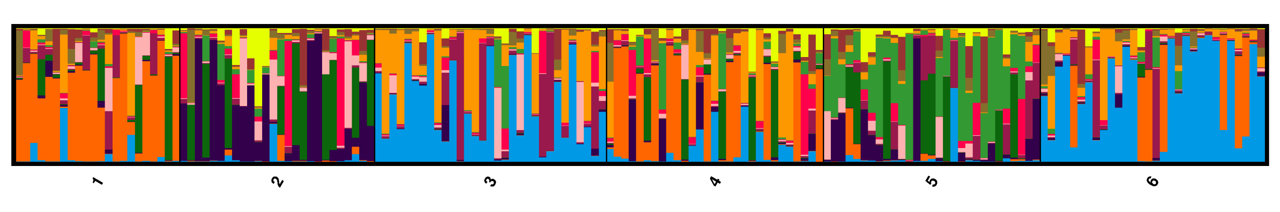

K=13 MinorCluster1

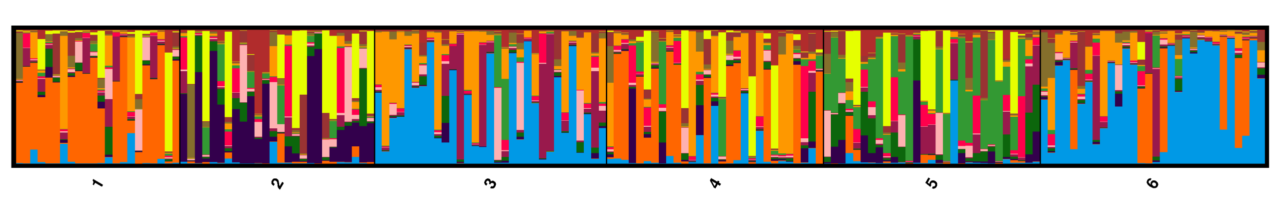

K=14 MinorCluster1

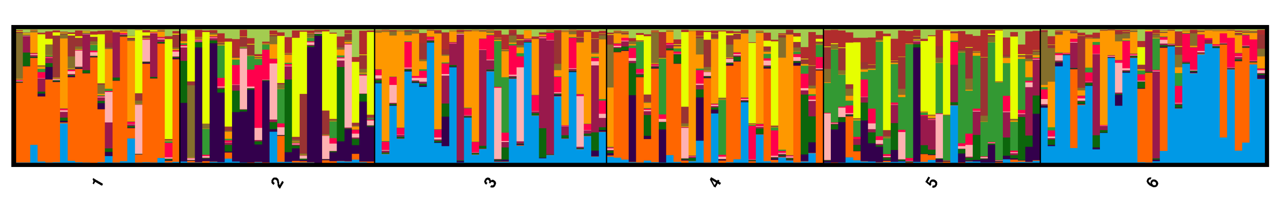

K=15 MinorCluster1

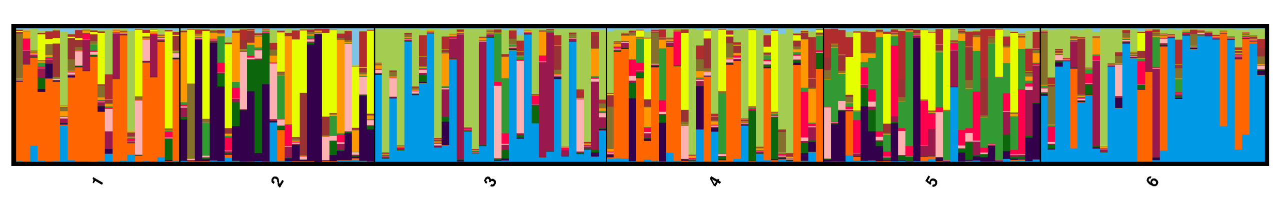

Division of runs by mode:

K=1	10/10
K=2	10/10
K=3	10/10
K=4	10/10
K=5	9/10, 1/10
K=6	6/10, 2/10, 2/10
K=7	5/10, 5/10
K=8	7/10, 3/10
K=9	5/10, 3/10, 2/10
K=10	8/10, 2/10
K=11	6/10, 2/10, 2/10
K=12	6/10, 2/10, 2/10
K=13	7/10, 3/10
K=14	6/10, 4/10
K=15	9/10, 1/10

CLUMPAK main pipeline - Job 1555298033 summary

Major modes for the uploaded data:

K=1

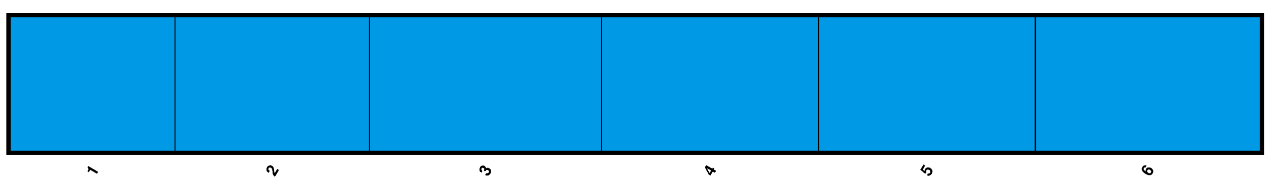

K=2

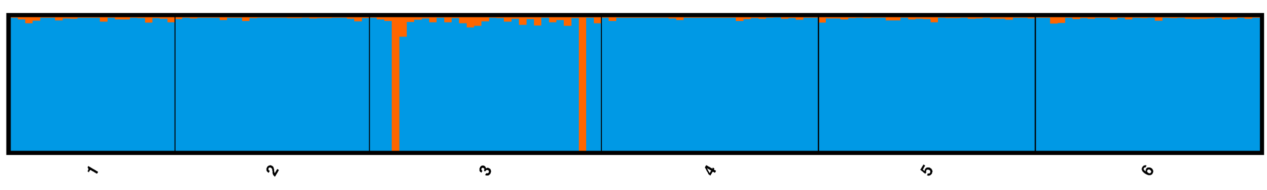

K=3

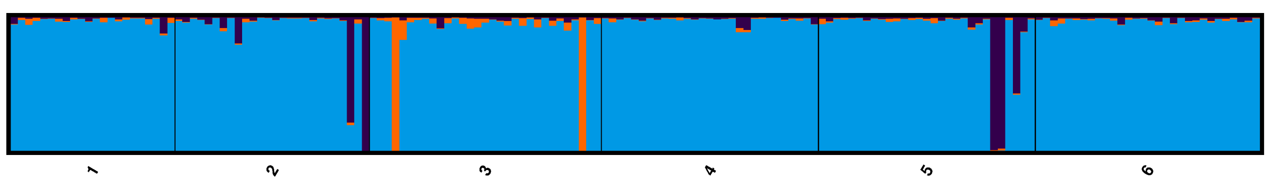

K=4

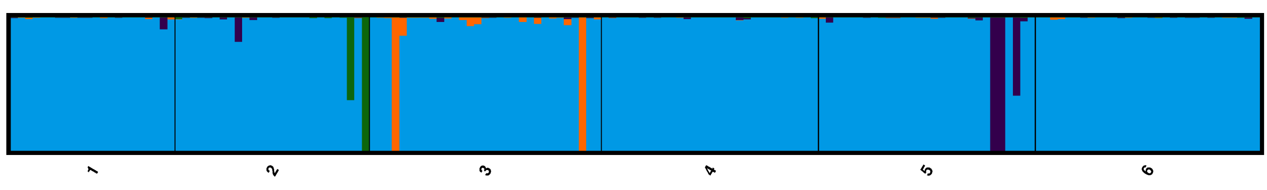

K=5

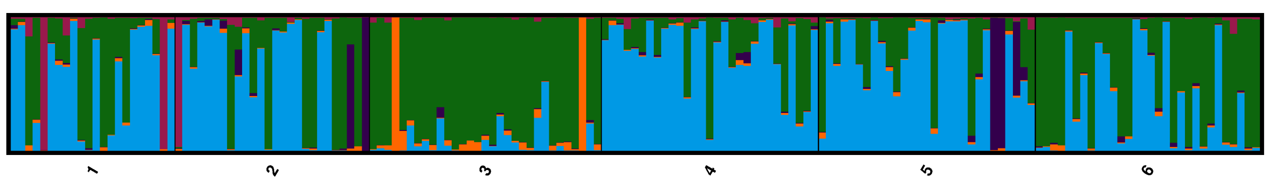

K=6

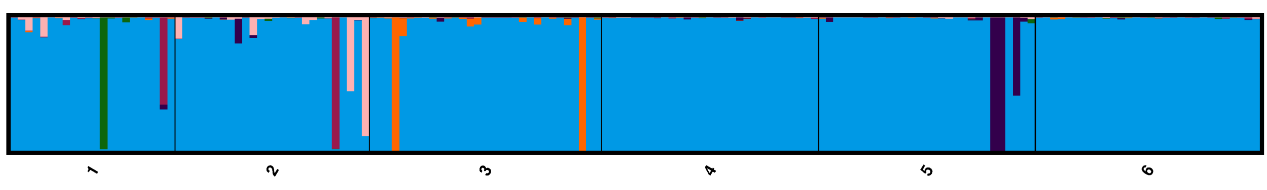

K=7

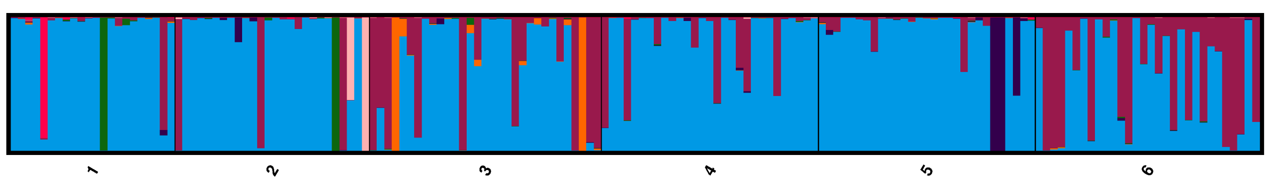

K=8

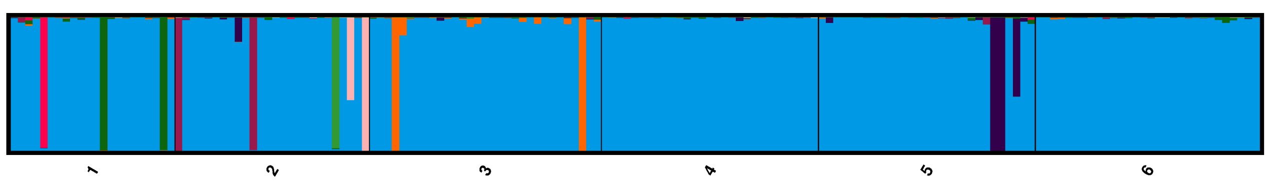

K=9

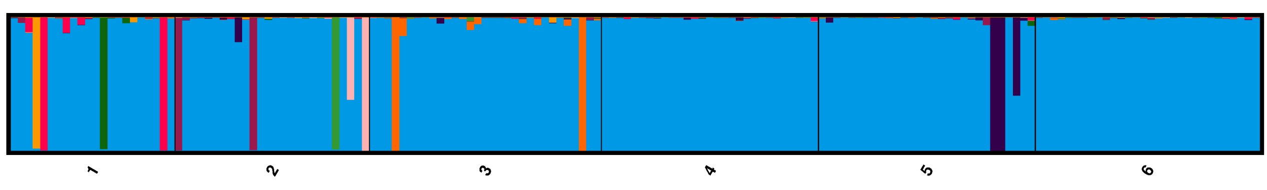

K=10

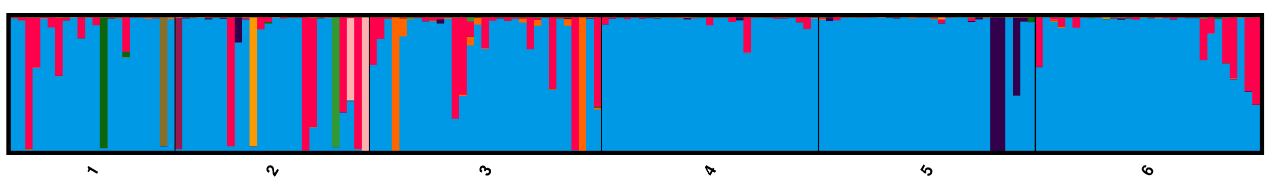

K=11

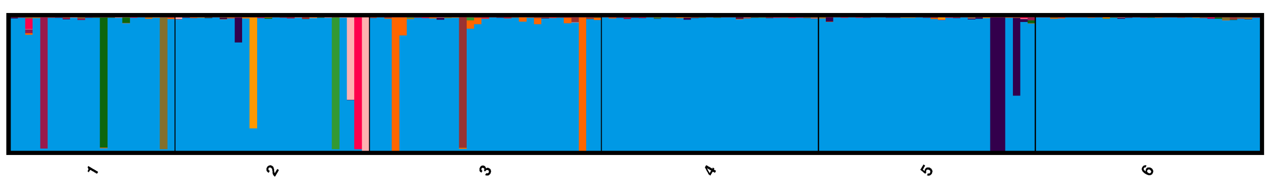

K=12

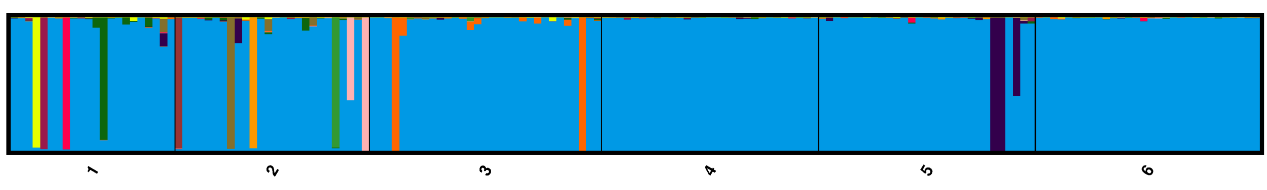

K=13

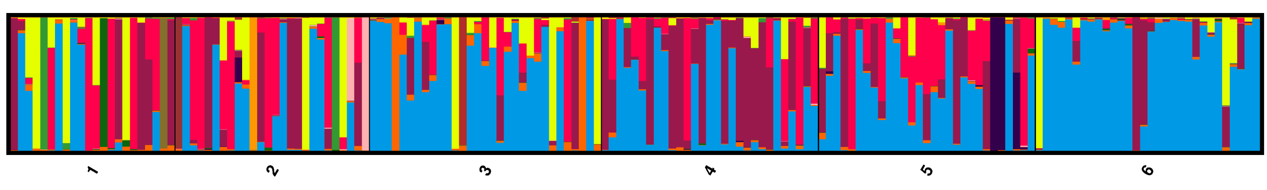

K=14

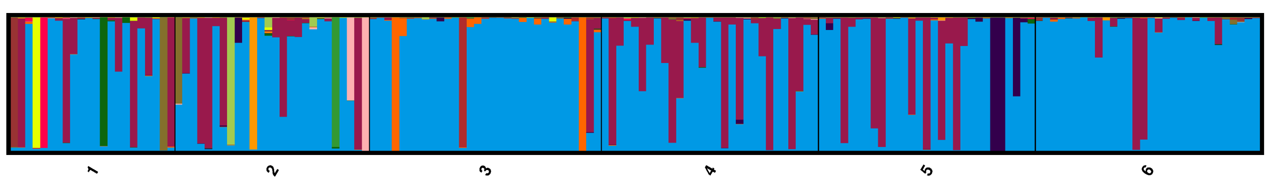

K=15

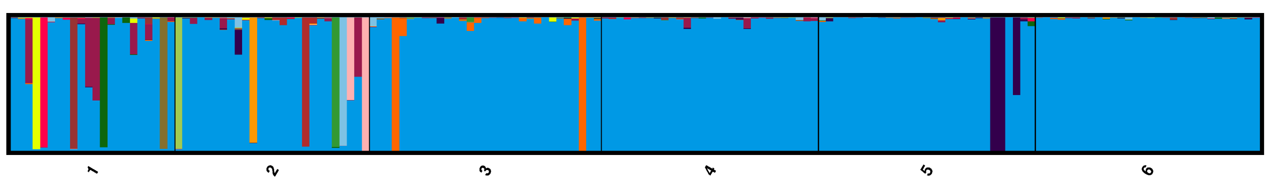

Minor modes for the uploaded data:

Division of runs by mode:

K=1	1/1
K=2	1/1
K=3	1/1
K=4	1/1
K=5	1/1
K=6	1/1
K=7	1/1
K=8	1/1
K=9	1/1
K=10	1/1
K=11	1/1
K=12	1/1
K=13	1/1
K=14	1/1
K=15	1/1
