# Supplementary material for: Fine-scale population structure and ecotypes of anadromous Hilsa shad (Tenualosa ilisha) across complex aquatic ecosystems revealed by NextRAD genotyping
Source: Sci Rep. 2019 Nov 5;9:16050. doi: 10.1038/s41598-019-52465-2 (PMC6831668; doi:10.1038/s41598-019-52465-2)
Supplement: Supplementary file 2 — Supplementary Info [file 41598_2019_52465_MOESM2_ESM.docx]

**Fine-scale population structure and ecotypes of anadromous Hilsa shad (*Tenualosa ilisha*) across complex aquatic ecosystems revealed by NextRAD genotyping**

Md Asaduzzaman^1^, Md A. Wahab^2^, Md J. Rahman^2^, Md Nahiduzzzaman^2^, Malcom W. Dickson^2^, Yoji Igarashi^3^, Shuichi Asakawa^3^ and Li Lian Wong^4, 5^*

^1^Department of Marine Bioresource Science, Faculty of Fisheries, Chattogram Veterinary and Animal Sciences University, Khulshi 4225, Chattogram, Bangladesh

^2^WorldFish, Bangladesh and South Asia Office, Banani, Dhaka, 1213, Bangladesh

^3^Laboratory of Aquatic Molecular Biology and Biotechnology, Department of Aquatic Bioscience, The University of Tokyo, 1-1-1 Yayoi, Bukkyo-ku, Tokyo 113-8657, Japan

^4^Institute of Tropical Aquaculture, Universiti Malaysia Terengganu, 21030 Kuala Terengganu, Malaysia

^5^Institute of Marine Biotechnology, Universiti Malaysia Terengganu, 21030 Kuala Terengganu, Malaysia

^*^Corresponding author

Dr. Li Lian Wong

Phone: 609-668-3671

Fax: 609-668-3390

Email: [lilian@umt.edu.my](mailto:liuzhan@auburn.edu)


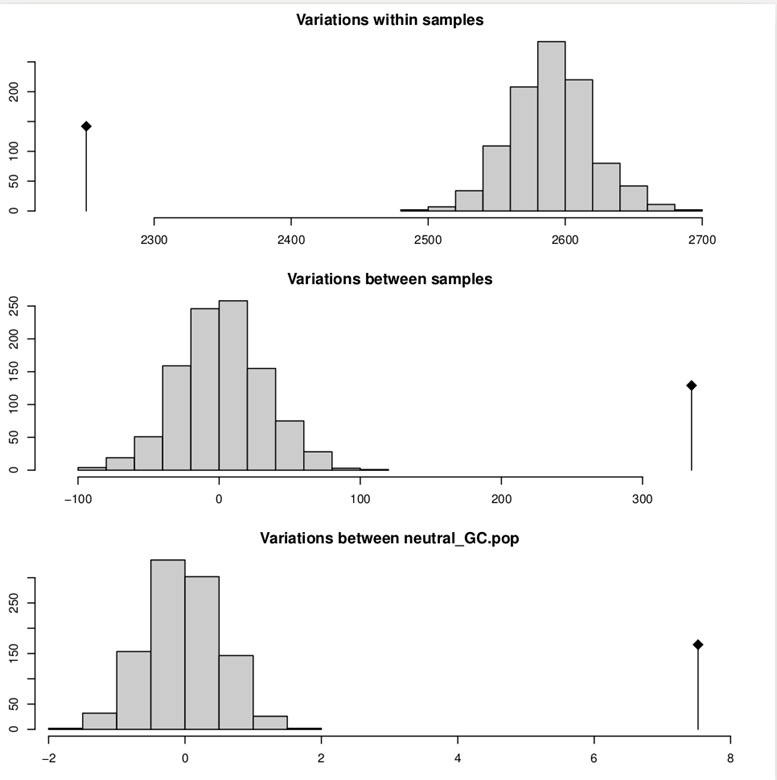

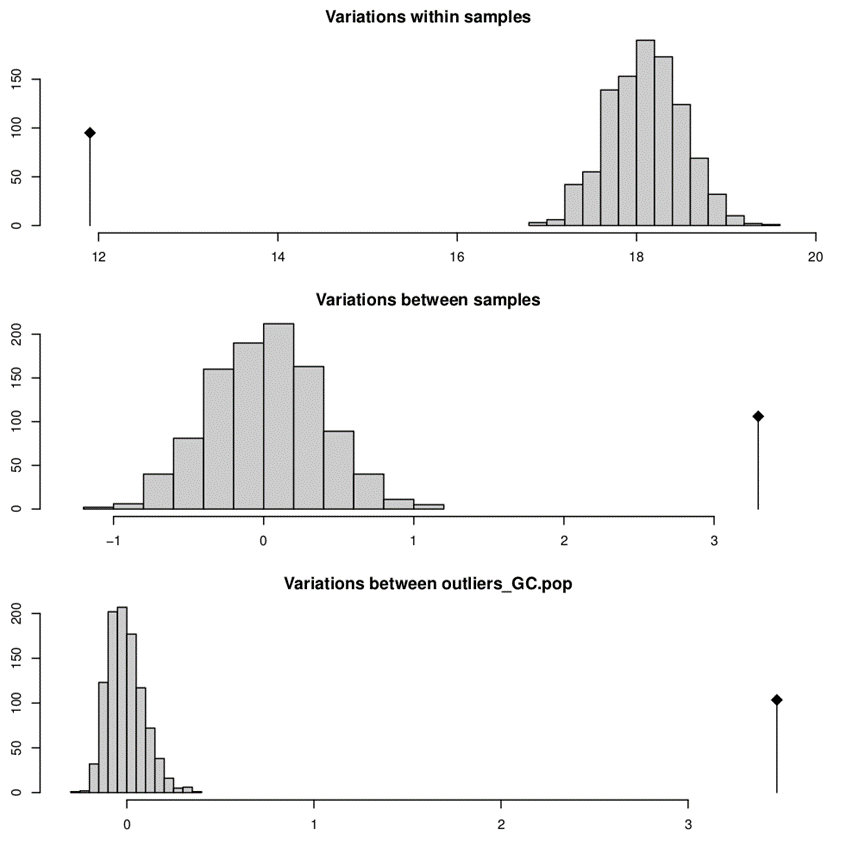


A

B

**Figure S1.** Histograms representing the distribution of the randomized strata based on the AMOVA result of the (A) putatively neutral SNP loci’s and (B) putatively adaptive SNP loci’s. The single data point represents the observed data and the bars represent a histogram of randomized results.  In the absence of any population structure the observed data point should fall within the distribution of randomized results. The above graphs showed significant population differentiation for both the neutral dataset and the adaptive dataset.

**Table S1**: Delta K value of each cluster calculated by ^35^.

| **K** | **Reps** | **Mean LnP(K)** | **Stdev LnP(K)** | **Ln'(K)** | **\|Ln''(K)\|** | **Delta K** |
| --- | --- | --- | --- | --- | --- | --- |
| 1 | 10 | -13158.40 | 0.43 | — | — | — |
| 2 | 10 | -10661.05 | 3.30 | 2497.35 | 1205.72 | 365.86 |
| 3 | 10 | -9369.42 | 1.69 | 1291.63 | 1084.03 | 640.06 |
| 4 | 10 | -9161.82 | 3.36 | 207.60 | 73.15 | 21.79 |
| 5 | 10 | -9027.37 | 39.10 | 134.45 | 25.52 | 0.65 |
| 6 | 10 | -8918.44 | 28.84 | 108.93 | 54.00 | 1.87 |
| 7 | 10 | -8863.51 | 78.75 | 54.93 | 53.10 | 0.67 |
| 8 | 10 | -8755.48 | 54.08 | 108.03 | 186.15 | 3.44 |
| 9 | 10 | -8833.60 | 419.28 | -78.12 | 246.12 | 0.59 |
| 10 | 10 | -8665.60 | 49.23 | 168.00 | 326.28 | 6.63 |
| 11 | 10 | -8823.88 | 556.06 | -158.28 | 368.12 | 0.66 |
| 12 | 10 | -8614.04 | 35.57 | 209.84 | 226.91 | 6.38 |
| 13 | 10 | -8631.11 | 102.23 | -17.07 | 19.80 | 0.19 |
| 14 | 10 | -8667.98 | 197.91 | -36.87 | 112.38 | 0.57 |
| 15 | 10 | -8592.47 | 88.10 | 75.51 | — | — |
